# MSCs-laden injectable self-healing hydrogel for systemic sclerosis treatment

**DOI:** 10.1016/j.bioactmat.2022.01.006

**Published:** 2022-01-19

**Authors:** Min Nie, Bin Kong, Guopu Chen, Ying Xie, Yuanjin Zhao, Lingyun Sun

**Affiliations:** a Department of Rheumatology and Immunology, Institute of Translational Medicine, The Affiliated Drum Tower Hospital of Nanjing University Medical School, Nanjing, 210002, China; b Department of Neurosurgery, Health Science Center, The First Affiliated Hospital of Shenzhen University, Shenzhen Second People's Hospital, Shenzhen, 518035, China; c State Key Laboratory of Quality Research in Chinese Medicines, Macau University of Science and Technology, Taipa, Macau (SAR), China; d State Key Laboratory of Bioelectronics, School of Biological Science and Medical Engineering, Southeast University, Nanjing, 210096, China

**Keywords:** Mesenchymal Stem cell, Injectable, Self-healing, Hydrogel, Systemic sclerosis

## Abstract

As a novel cellular therapy, the anti-inflammatory and immunomodulatory virtues of mesenchymal stem cells (MSCs) make them promising candidates for systemic sclerosis (SSc) treatment. However, the clinical efficacy of this stratagem is limited because of the short persistence time, poor survival, and engraftment of MSCs after injection *in vivo*. Herein, we develop a novel MSCs-laden injectable self-healing hydrogel for SSc treatment. The hydrogel is prepared using N, O-carboxymethyl chitosan (CS-CM) and 4-armed benzaldehyde-terminated polyethylene glycol (PEG-BA) as the main components, imparting with self-healing capacity via the reversible Schiff-base connection between the amino and benzaldehyde groups. We demonstrate that the hydrogel laden with MSCs not only promoted the proliferation of MSCs and increased the cellular half-life *in vivo*, but also improve their immune-modulating functions. The tube formation assay indicates that the MSCs could significantly promote angiopoiesis. Moreover, the MSCs-laden hydrogel could inhibit fibrosis by modulating the synthesis of collagen and ameliorate disease progression in SSc disease model mice after subcutaneous injection of bleomycin. All these results highlight this novel MSCs-laden hydrogel and its distinctive functions in treatment of chronic SSc, indicating the additional potential to be used widely in the clinic.

## Highlights

• We proposed novel MSCs-laden injectable self-healing hydrogel for SSc treatment.
• The hydrogel was constructed by PEG-BA and CS-CM.
• MSCs-laden hydrogel promotes angiopoiesis and inhibit fibrosis.

## Graphical abstract

**Figure:**
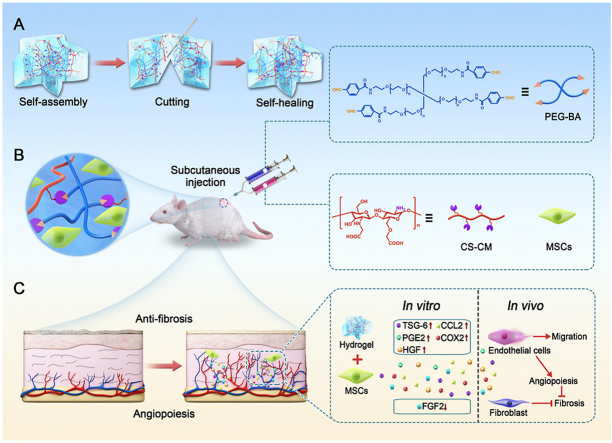

## 1 Introduction

Systemic sclerosis (scleroderma, SSc), a considered incurable immune-mediated rheumatic disease, is characterized by complex interplays between vasculopathy, inflammation, immunological abnormalities and progressive fibrosis [1,2]. The traditional therapies are combinations of immunosuppressants, such as mycophenolate mofetil or cyclophosphamide, these immunosuppressants can be supplemented by targeted biological and antifibrotic therapies, even hematopoietic stem cell transplantation are used for the treatment of refractory SSc, no therapy has so far been able to slow or modify the natural progression of this disease [[3], [4], [5], [6], [7], [8], [9]]. Multipotent mesenchymal stem cells (MSCs) therapy with demonstrated remarkable improvement has gradually emerged as a promising therapeutic candidate [[10], [11], [12], [13], [14]]. MSCs are multipotent cells that can be expanded from various adult and perinatal tissues. In addition, it has been suggested that MSCs have a minimal risk of initiating an allogeneic immune response when injected *in vivo* [11,15]. Moreover, MSCs display profound immunosuppressive effects on various immune cells and inherent trophic activity, anti-inflammatory properties and immunomodulatory characteristics, especially by secreting a large panel of bioactive molecules to address the most important key points of the refractory SSc [13,[16], [17], [18], [19], [20]]. MSCs and MSCs-derived extracellular vesicles therapy have shown efficacy in several animal models of SSc including HOCl-induced and bleomycin-induced SSc model [[21], [22], [23]]. However, the clinical application of MSCs therapy is still challenged, because their suboptimal encapsulation and injection approaches lead to poor survival, retention time and engraftment [24,25]. Therefore, novel MSCs therapy with high survival, increased retention, and curative potentials are need to be improved for treatment of SSc.

**Fig. 1:**
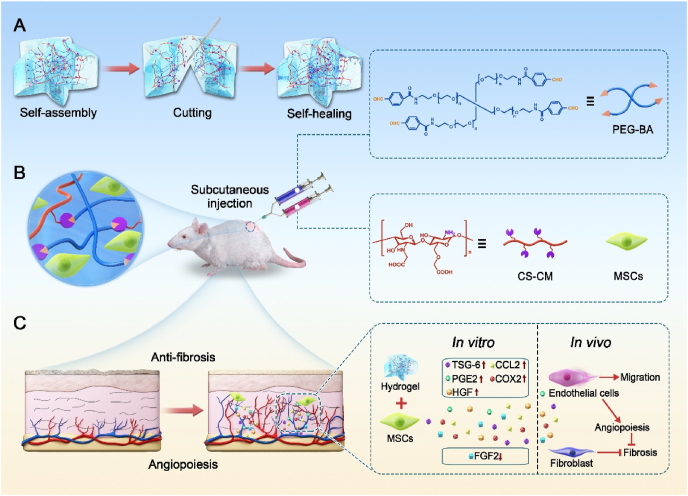
Schematic demonstrating the fabrication of MSCs-laden injectable self-healing hydrogel for systemic sclerosis (SSc) treatment after subcutaneous injection in SSc mice. (A) Schematic diagram of self-healing process of hydrogel. (B) Reaction scheme to show crossing-linking of PEG-BA and CS-CM via the Schiff-base reaction and the application in bleomycin induced SSc disease model mice. (C) Schematic diagram for investigation of the paracrine products generated by MSCs under the influence of hydrogel to modulate the cell communication network toward anti-fibrosis.

In this study, we proposed a novel MSCs-laden injectable self-healing hydrogel for SSc treatment, as schemed in Fig. 1. Hydrogels are three-dimensional (3D) cross-linked network structural substances characterized by bio-inspiration, bio-functionality, and biomimicry, resulting in variable physicochemical performances [[26], [27], [28], [29], [30], [31]]. Due to their high-water contents and regulatory mechanical capacities, cell-laden hydrogels as biomimetic tissues are considered as promising minimally invasive injection carrier through percutaneous needles [[32], [33], [34], [35], [36], [37], [38]]. Among these hydrogels, polyethylene glycol, chitosan and their derivatives are favorable owing to their low price, non-immunogenicity, easy synthesis, bioactivity, good degradability *in vivo* and inherent biocompatibility [[39], [40], [41], [42], [43], [44]]. However, their low solubility in most solvents and restricted chemical modification methods hinder their further application as injectable hydrogels [45]. Therefore, novel biomedical hydrogels with suitable controllability in gelation time, mechanical strength and self-healing capacity by modifying the functional groups and modulating the concentrations of gelling components and their mass ratios to enhance the clinical efficacy of MSCs therapies is still highly anticipated. In addition, although hydrogel materials have been widely used in many aspects of biomedical areas [[46], [47], [48], [49]], to the best of our knowledge, research on the treatment of SSc with this versatile hydrogel laded with MSCs has never been reported.

Herein, we developed the desired MSCs-laden injectable self-healing hydrogel by using N, O-carboxymethyl chitosan (CS-CM) and 4-armed benzaldehyde-terminated polyethylene glycol (PEG-BA) as main components. CS-CM was synthesized by carboxymethylation of chitosan (CS), and meanwhile, PEG-BA was prepared by condensation of 4-arm-amino-terminated PEG with 4-formylbenzoic acid (4-BA). The 3D hydrogel network was constructed and endowed with self-healing capacity via the reversible Schiff-base connection between the amino and benzaldehyde groups. Self-healing hydrogels are a kind of smart hydrogel that can be injected without gel fragmentation and maintain integrity at the target site due to the ability to heal damage automatically, avoiding the risk of skin structure damaging from premature polymerization. Furthermore, bare MSCs may be due to the degradable formulation which may lose their physical integrity more quickly once exposed to proteases. Therefore, formation of MSCs-laden hydrogel could promote cell viability and survival. We revealed that the MSCs-laden hydrogel not only promoted MSCs proliferation and increased the cellular half-life *in vivo*, but also improved their immune modulating functions. These features make the MSCs-laden hydrogel valuable in chronic SSc treatment and promising for various disease treatments.

## 2 Materials and methods

### 2.1 Materials

Chitosan (2 × 10^5^ Da), sodium periodate, monochloroacetic acid and ethylene glycol were purchased from Sigma-Aldrich. PEG-BA (Mw 6000) was purchased from Ponsure Biotechnology. Cell counting kit-8 (CCK8) assay kit was obtained from KeyGEN BioTECH Co., Ltd (Nanjing, China). Calcein-AM/PI Double Stain Kit was obtained from Yeasen Biotechnology Co., Ltd (Shanghai, China). FITC-conjugated anti-human CD14, CD19, CD45, CD34, PE-conjugated anti-human CD73, CD105, CD90, HLA-DR antibodies were obtained from BD Biosciences (San Jose, CA). All of the antibodies including α-SMA, vimentin, PLIN1, COL1 were purchased from Cell Signaling Technology.

### 2.2 Synthesis and characterization of PEG-BA and CS-CM

Synthesis of CS-CM was based on a previously reported procedure with some modification [50]. 10 mL of isopropyl alcohol was employed to suspend 1 g CS and stirred at RT. Afterwards, 5 mL of sodium hydroxide solution (10 mol/L) was dropwise added into the above solution and stirred for 30 m. 10 mL of monochloroacetic acid was added into five equal portions. The solution was stirred for 3 h. Hydrochloric acid solution (10%) was employed to adjust pH of the reaction mixture to about 7.4. The CS-CM was washed with methanol and alcohol. A freeze drier was used to get the dry product.

The hydrogels were obtained by mixing the PEG-BA and CS-CM solutions. Briefly, an 8% (w/v) PEG-BA solution was prepared by dissolving product in cell culture medium. A 3% (w/v) CS-CM solution was prepared by using cell culture medium as solvent too. Subsequently, the dynamic frequency sweep rheological test was processed on a Discovery HR-2 rheometer. After CS-CM and PEG-BA solutions were mixed on the parallel plate, *G*′ and *G*″ were collected.

The equivalent volume of CS-CM solution stained with trypan blue and PEG-BA solution dyed with rhodamine B were filled into a dual syringe system. The precursor solutions in syringes passed through the 26-gauge needle easily without blocking, followed by the instantaneous formation of hydrogels. Subsequently, two pieces of flower-shaped hydrogels stained by rhodamine B and methylene blue, were cut into equal two parts, respectively. After 20 min at 25 °C, the total 4 pieces of alternate colors were combined into two integral blended flower-shaped pieces. Self-healing was confirmed by the capacity of the healed flower-shaped hydrogel to hold its structure when suspended under gravity.

### 2.3 Clinical-grade MSCs and condition medium collection

From the separation, culture and identification processes were conformed with the quality standards. MSCs with an initial density of 1 × 10^4^ cells/cm^2^ were cultured in 75 cm^2^ cell culture flasks at 37 °C containing 5% CO_2_ in DMEM/F12 with 10% FBS for 48 h. Condition medium was harvested and centrifuged at 1500 rpm for 5 min and used for experiments.

### 2.4 Prepare of MSCs laden hydrogel and in vivo residence time

Cell viability of MSCs was assessed after incubation in hydrogel extracts with different concentrations (0, 5,10, 20 mg/mL) for 1 and 3 days. After incubation, Cell Counting Kit-8 was used according to the manufacture's instruction. For cell encapsulation, cell suspensions of MSCs were mixed with PEG-BA (8 wt%) and CS-CM (2 wt%) solutions. Then, 1 mL of the mixture was pipetted into a 24-well culture plate. Samples were imaged using a confocal microscope every day for three days.

IVIS was applied to investigate the hydrogel enhance retention of MSCs in tissue. an *in vivo* experiment was carried out after subcutaneously injection of luciferase-labeled MSCs in BALB/c mice. The IVIS and luminescent signal analysis was performed as described [51].

### 2.5 Wound healing assays and in vitro tube formation experiment

HUVECs were used in wound healing experiment and tube formation assay. After scratching the cell monolayer with a pipette, HUVECs were cultured with the supernatant from MSCs or the cell culture medium, and representative images were taken after incubation for 24 and 48 h for tube formation experiment, 1 × 10^4^ HUVECs were seeded in 48-well plate with adding 100 μL of growth factor-reduced Matrigel (BD Bioscience). Following incubation with condition medium and culture medium, confocal microscope was applied to take images.

### 2.6 QRT--PCR and RNA sequencing

Total RNA from cultured cells in cell culture plate, hydrogel, and mouse skin samples were extracted using Trizol reagent (Vazyme Biotech, China). QRT-PCR was performed using a FastStart Universal SYBR Green Master (Vazyme Biotech, China) in a StepOne (Thermo Fisher). The primer sequences for qRT-PCR were listed in Supplementary Table 1.RNA-seq analysis was conducted by Novogene Co., LTD (Beijing, China).

### 2.7 *In vivo* degradation and toxicity behavior of the hydrogel

BALB/c mice were employed to evaluate the *in vivo* degradation and toxicity property of the hydrogel. 0.4 mL of the hydrogels were administered by dorsal subcutaneous injection. Three mice from each group were sacrificed at 5min,1d, 3d, 5d respectively. The injection sites were opened with a surgical scissors to observe the state of hydrogels. In order to determine whether hydrogel had potential toxicity, the hydrogels were subcutaneously injected into the dorsal side of mice for 21 days. Important organ samples including heart, liver, spleen, lung and kidney were sent for histopathological examination for toxicity evaluation.

### 2.8 Animals and treatment

All animal care and handing procedures were conformed with the ethics (ethics number 2020AE01039). To induce skin fibrosis, mice were conditioned by subcutaneous injections with 1 mg/mL bleomycin (Aladdin, Shanghai, China) in saline at a dose of 100 μL in each mouse every day for 4 consecutive weeks. One group of the mice were received with 100 μL PBS solution. The other three groups mice were treated with hydrogel, MSCs and MSCs-loaded hydrogel (Hy-MSCs) at a dose of 5 × 10^6^ cells or hydrogel by subcutaneous injections. BALB/c mice were used as control group. Mice were sacrificed at day 10 to collect skins for subsequent experiments.

### 2.9 H&E, masson staining and immunofluorescence

After skins isolation and fixation in paraformaldehyde, the tissues were embedded in paraffin for H&E and Masson staining. The skin tissues were cut for 7 μm for immunohistochemistry. The antibodies were used as follows: α-SMA, COL1, and PLIN1.

### 2.10 Statistical analysis

Statistical analysis was performed by GraphPad Prism software. All data were presented as average ± s.d. Student's t-test was used for comparison between groups.

## 3 Results and discussion

**Fig. 2:**
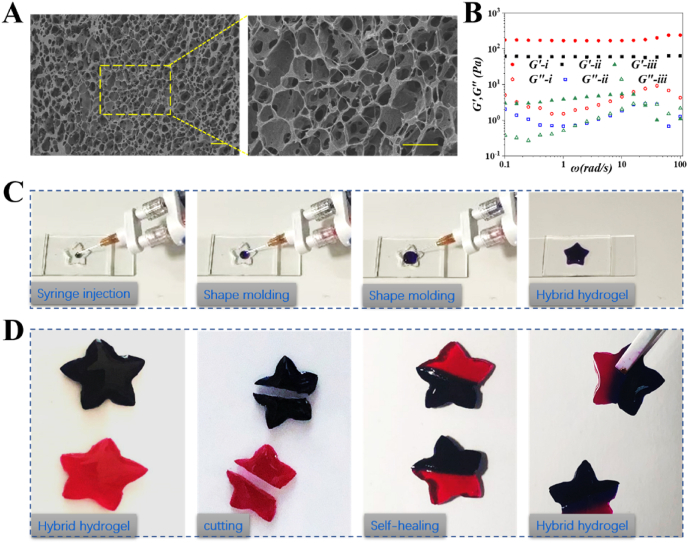
Characterization of the hydrogel. (A) SEM analysis of the hydrogel structure. The scale bar is 10 μm. (B) Rheological properties of the hydrogel at room temperature (RT). (C) Injectable capability of the hydrogel. (D) Self-healing properties of the hydrogel at RT.

CS-CM was normally synthesized by covalently modifying CS with acetic acid, whereas PEG-BA was formed by the condensation of amino-terminated PEG with 4-BA (Figure S1). The hydrogel was prepared by mixing of CS-CM and PEG-BA solution. The mechanical capacities of hydrogel are directly related to their microscopic structures [52]. Scanning electron microscopy (SEM) revealed that hydrogels had a highly interconnected porous structure. (Fig. 2A).

Meanwhile, the mechanical strength of the hydrogel under different ratios of two components was studied by the dynamic frequency sweep rheological test. Hydrogels were formed with uniform concentration of PEG-BA (4 wt%) and three different concentrations of CS-CM (1.5 wt%, 1 wt%, 0.5 wt%), the molar ratios of - NH_2_ and –CHO are 1.5:1, 1:1 and 0.5:1, and As Fig. 2B showed, the hydrogels provided the frequency-independent gelation characteristics, and the storage modulus *G*^*’*^ far surpassed the loss modulus *G*^*’’*^ at a shear frequency ranging from 0.1 to 100 rad/s, indicating that the hydrogel behaved as an elastic solid. Moreover, the value of *G*^*’*^ and *G*^*’’*^ can be regulated by changing the ratio of gelling elements. As expected, higher storage modulus *G*^*’*^ value was observed at high concentration of CS-CM.

Appropriate injectable property of hydrogel is necessary for their biomedical application. To evaluate the injectability of the hydrogel, CS-CM solution stained with trypan blue and PEG-BA solution dyed with rhodamine B were filled into a dual syringe system at an equivalent volume. The precursor solutions in syringes easily passed through the 26-gauge needle without blocking, and then forms hydrogels immediately (Fig. 2C). Further, the hydrogel can be moulded into any shape, providing a broad application scope in clinical practice.

The self-healing property was evaluated by a macroscopic test of two pre-gelled flower-shaped hydrogels with blue (trypan blue) and red (rhodamine B) colors. As Fig. 2D showed the cut hydrogel blocks could be recombined into the cmmplete flower-shaped hydrogel without any external stimulation, and the self-healing hydrogel had enough mechanical strength to be lifted.

Appropriate degradation properties of hydrogels are necessary for their biomedical application. To investigate the degradation feature, the hydrogels were subcutaneously injected into the dorsal side of mice, three mice from each group were sacrificed at 5 min, 1 d, 3 d, 5 d respectively. It was found that the amount of hydrogel decreased gradually in 5 days as a result of the existence of lipase, protease, and hyaluronidase in the mouse body (Figure S2). In order to determine whether hydrogel had potential toxicity, the hydrogels were subcutaneously injected into the dorsal side of mice for 21 days. Important organ samples including heart, liver, spleen, lung and kidney were sent for histopathological examination, which revealed no significant pathological changes (Figure S3). Therefore, the hydrogel with adjustable mechanical capability, good injectability, self-healing, degradation and non-toxicity property will be valuable in the regulation of the translation fates of stem cell in MSCs therapies.

The hydrogel was subsequently encapsulated with MSCs as a cell therapy for SSc treatment. First, clinical-grade MSCs were isolated from human umbilical cord, and their quality such as morphology, phenotype, and differentiation potential were evaluated. MSCs are plastic-adherent and able to differentiate into the osteogenic and adipogenic properties (Figure S4A, B). They are distinguished from other types of cell by expressing cell-surface markers CD73, CD105 and CD90, as well as positive for mesenchymal markers (vimentin and α-SMA) (Figure S4D), but lack the expression of CD34, CD19, HLA-DR, CD45 and CD14 (Figure S4C).

It is well known that cytocompatibility is the basic requirement for hydrogel as cell carriers. Therefore, cell viability of MSCs was assessed after incubation in hydrogel extracts with different concentrations (0, 5,10, 20 mg/mL) for 1 and 3 days. As Figure S5 A，B showed，there was no remarkable difference between the treatment and the control group in cell viability. The fluorescent results of live cells (Figure S4 C) are also consistent with the quantitative viability. These results suggested that the cytotoxicity of developed hydrogel to MSCs was negligible, thus confirming the remarkable cytocompatibility of CS-CM/PEG-BA hydrogel for using as stem cell carrier.

**Fig. 3:**
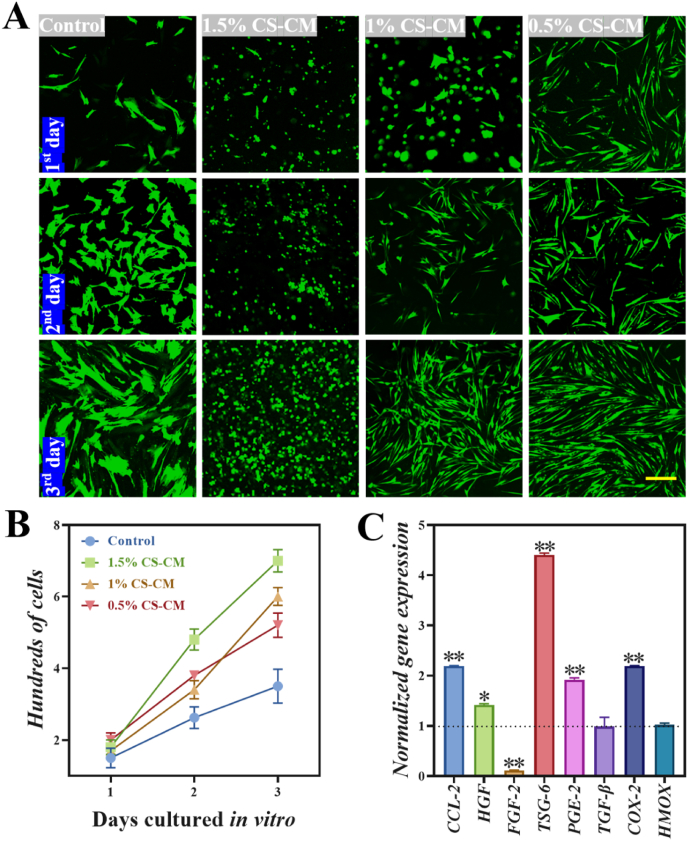
Hydrogel facilitated MSCs cellular morphology, proliferation and immunomodulatory function *in vit*ro. (A) Fluorescent images showed the MSCs proliferation during three days culture in tissue culture or in hydrogel. The scale bar is 200 μm. (B) Cells proliferated within the hydrogel and compared with the cells in tissue culture flask. (C) Gene expression of cytokines produced by MSCs in hydrogel which was normalized to that of MSCs on tissue culture. *P < 0.05, **P < 0.01.

Moreover, hydrogels should support cell growth and network formation. We developed an *in vitro* MSCs cell morphology and proliferation model. MSCs suspension incorporated with CS-CM/PEG-BA hydrogels at three different concentrations of CS-CM (1.5 wt%, 1 wt%, 0.5 wt%) for three days were compared with control group cultured in tissue culture flask. As shown in Fig. 3A, MSCs exhibited continuous proliferation during the three-day culture period. In contrast, the control group showed growth inhibition when the cells covered the flask. Moreover, MSCs continued to proliferate and avoided the growth inhibition during 3D culture in hydrogels. Notably, the cells grown in the hydrogels with uniform 4 wt% PEG-BA and different concentrations of CS-CM (1.5 wt%, 1 wt%, 0.5 wt%) maintained viability. In the 1.5% CS-CM group, we noticed that most MSCs were still round and oval, even after culturing for three days as shown in Fig. 3A. However, cells in the 1% and 0.5% CS-CM groups began to exhibit a long shuttle-shaped spread morphology, which continued for three days. The 1% CS-CM group was particularly noteworthy because the shorter gelation time and cells rapid proliferation rate, indicating that MSCs in this group were able to evenly disperse and form highly interconnected cellular networks compared to 0.5% CS-CM group (Fig. 3B). Given these obvious advantages, 1% CS-CM was selected in our study.

In addition, MSCs secret multifunctional molecules via paracrine mechanisms to exert their immunomodulatory capacities. The secretome is a multifaceted group of cytokines, chemokines and growth factors, such as cyclooxygenase 2 (COX-2), hepatocyte growth factor (HGF), tumor necrosis factor-stimulated gene 6 protein (TSG-6), fibroblast growth factor 2 (FGF-2), prostaglandin E synthase 2 (PGE-2), heme oxygenase (HMOX), C–C motif chemokine 2 (CCL-2) and transforming growth factor-β (TGF-β), many of which depend on the target cells and the microenvironment surrounding the cells [13,17,53]. We investigated the immunomodulatory function of MSCs in hydrogel by qRT-PCR. Analysis of the secretome of infused MSCs in the inflamed tissue microenvironment showed that it contained various cytokines, including TGS-6 and CCL-2. The elevation of TGS-6 and CCL-2 were the most remarkable among the selected factors examined, while the mRNA expression of TGS-6 and PGE2 of MSCs in hydrogels were about 4.5-fold and 2.2-fold higher than that of MSCs in tissue culture, respectively (Fig. 3C). COX-2 is a crucial enzyme for the synthesis of PGE-2, and PGE-2 is an important anti-inflammatory mediator secreted by MSCs. Compared with MSCs in tissue culture, the mRNA levels of the COX-2 and PGE-2 in hydrogel increased by about 2.2-fold and 2-fold, respectively. MSCs produce a series of growth factors such as HGF and FGF-2. Especially in the presence of inflammatory cytokines, the expression level of fibrosis factor FGF-2 was significantly decreased, while the angiogenic HGF was increased about 1.5-fold. As shown in Fig. 3C, after treating encapsulated MSCs, the expression of FGF-2 was reduced, while the levels of CCL-2, HGF, TSG-6, PGE-2 and COX-2 increased, indicating an enhanced immunomodulatory and angiogenesis function of MSCs therapy.

**Fig. 4:**
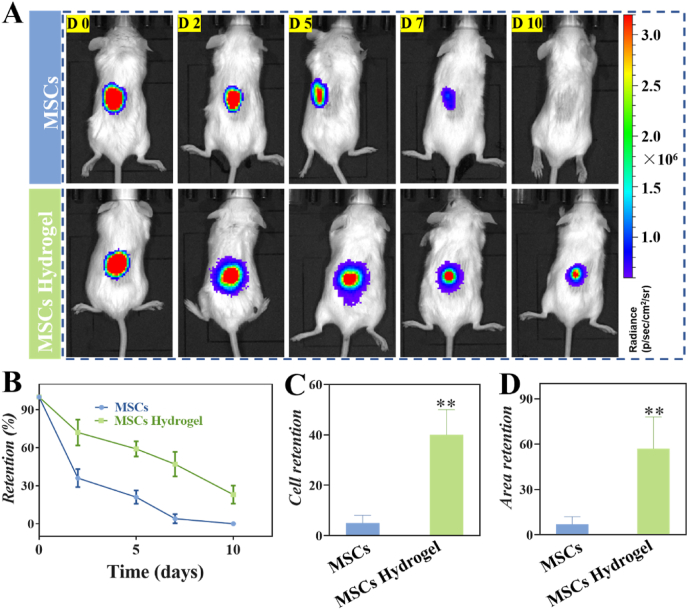
Hydrogels promote the retention of MSCs *in vivo*. (A) Representative luminescent signal images of MSCs that were subcutaneously injected of mice with and without hydrogel encapsulation at 0, 2, 5, 7, and 10 days. (B) The combined radiance at each time point. Comparison of cell persistence (C) and cell area (D) at day 7 relatively to day 0. **P < 0.01.

To investigate whether hydrogel enhance retention of MSCs in tissue, an *in vivo* experiment was carried out after subcutaneously injection of luciferase-labeled MSCs in BALB/c mice to recapitulate the survival of MSCs in the presence of a functional immune system. IVIS was applied to image the fluorescent intensity of the recipient animals at 0, 2, 5, 7 and 10 days (Fig. 4A). MSCs-laden hydrogels led to a longer *in vivo* retention time of MSCs compared to the bare MSCs group at the end of 10 days (Fig. 4B and C). Moreover, the cell area was also significantly higher in MSCs-laden hydrogel group compared to that of the bare MSCs group at the radiant efficiency above 2 × 10^7^ (Fig. 4D). The shorter *in vivo* residence time of bare MSCs may be due to the degradable formulation which may lose their physical integrity more quickly once exposed to proteases. Taken together, these results proved that the formation of MSCs-laden hydrogel promoted cell viability and survival, leading to the increment of immunomodulatory function and therapeutic effects *in vivo*.

**Fig. 5:**
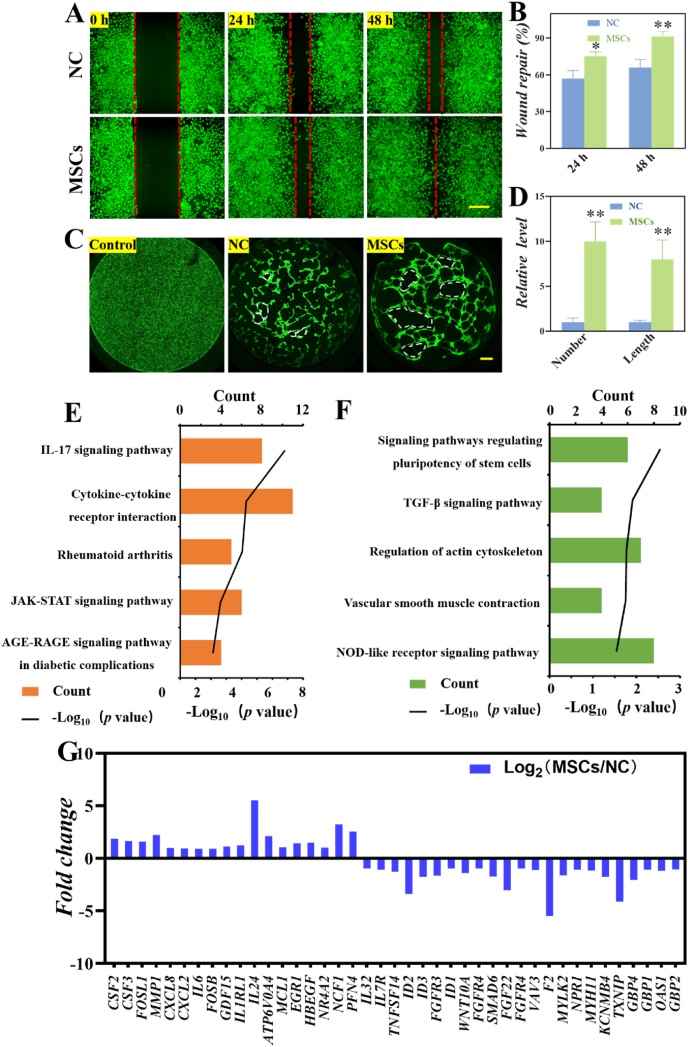
MSCs promoted HUVECs angiogenesis and migration *in vitro*. (A) Representative images of wound healing assay in medium (NC group) and conditional medium (MSCs group). (B) Wound healing assay results are quantified in the histogram. (C) Fluorescent images of HUVECs cultured in cell culture flask (Control group), cell culture flask covered by matrigel (NC group) and cell culture flask covered by matrigel in MSCs conditional medium (MSCs group). The scale bar is 200 μm. (D) Statistical analysis of total tube number and length in each group. *P < 0.05, **P < 0.01. KEGG analysis of top 5 enriched up-regulated (E) and down-regulated (F) terms in MSCs group compared with NC group. (G) The expression of markers genes in MSCs and NC groups.

SSc disease is characterized by a widespread vasculopathy of skin and internal organs. To investigate whether the cytokines and growth factors produced by MSCs can promote cell migration and angiogenesis, we first performed wound healing experiments using human umbilical vein endothelial cells (HUVECs). After incubated HUVECs with the culture supernatant from MSCs (MSCs group) or the cell culture medium (NC group), the migratory ability of HUVECs cultured with the supernatant from MSCs was increased compared to that cultured in the medium as the wound healing assay showed (Fig. 5A and B). Consistently, tube formation assay, showed that no tubular structures were formed when the HUVECs cultured in cell culture flask (Control group) as shown in Fig. 5C. The HUVECs cultured with MSCs supernatant developed more tubular structures than cultured in medium (NC group). We noticed that the total tube numbers and lengths in the MSCs group were more and longer than that those of the NC group in Fig. 5D. These results indicated that MSCs can produce cytokines and growth factors that promote angiogenesis. And, moreover, we showed that MSCs played a crucial role in modulating HUVECs migration and angiogenesis *in vitro*.

To explore the molecular mechanism of MSCs promoting angiogenesis of HUVECs, we performed RNA sequencing (RNA-seq) analysis to profile the transcriptome of HUVECs cultured with MSCs supernatant (MSCs group) in the flask covered by matrigel and normal medium (NC group). Compared with the NC group, there were 594 genes differentially expressed (*p*﹤0.001), of which 245 were increased and 349 were reduced (Figure S6). Kyoto Encyclopedia of Genes and Genomes (KEGG) pathway analysis indicated that the up-regulated genes in the MSCs treated group were enriched for functional annotations relating to IL-17 signaling pathway, cytokine-cytokine receptor interaction, rheumatoid arthritis, KAK-STAT signaling pathway and AGE-RAGE pathway. Whereas, KEGG analysis of top 5 down-regulated terms in MSCs group compared with NC group was related to signaling pathways regulating pluripotency of stem cells, TGF-β signal pathway, regulation of actin cytoskeleton, vascular smooth muscle contraction and NOD-like receptor signal pathways (Fig. 5E and F). Moreover, KEGG pathway analysis showed that the up-regulated genes in the MSCs treated group were enriched for functional annotations, involving the induced release of certain chemokines, cytokines, matrix metalloproteinases (MMPs), response to specific binding receptors on the target cell surface, and regulation of cell growth and angiogenesis. Furthermore, subsequent enrichment analysis revealed that the down-regulated genes in the MSCs group were associated with the pluripotency maintenance, the formation of tissues and organs, and the immune response. The expression of the marker genes in the five up-regulated and down-regulated pathways in HUVECs were shown in Fig. 5G. The network of KEGG pathways illustrating the interactions between the groups of genes was shown in Figure S7.

**Fig. 6:**
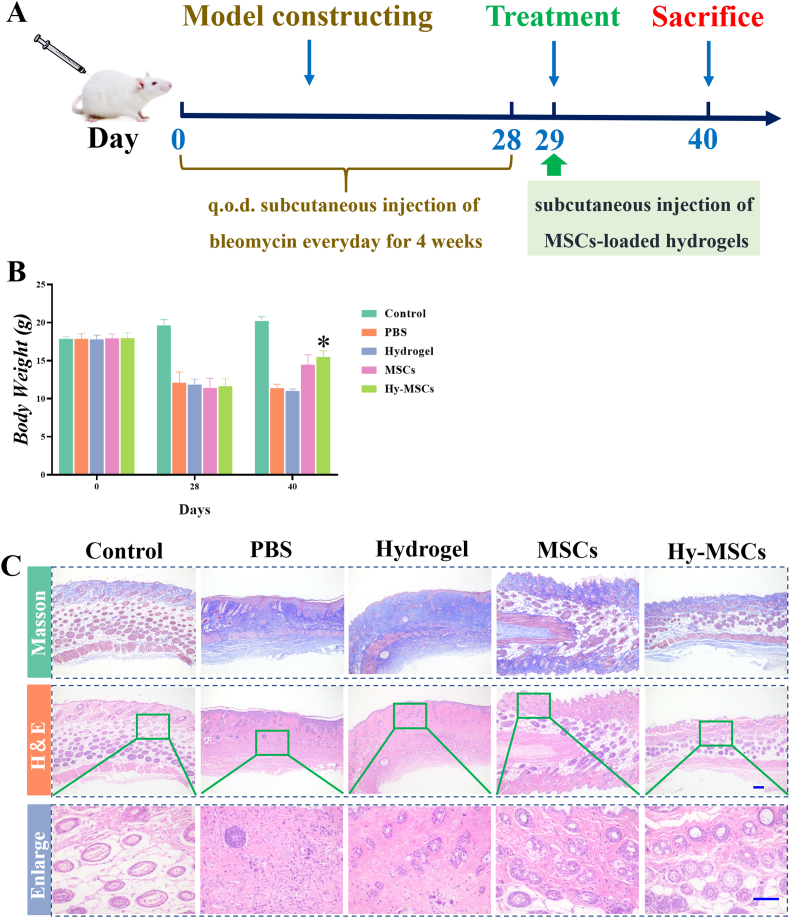
Schedule and comparative studies of therapeutic efficacy in bleomycin-induced SSc model mice. (A) Schedule of the establishment and treatment of the SSc mouse model. (B) Body weight of Control, PBS, hydrogel, MSCs, and Hy-MSCs groups at different time-points during the induction and treatment of bleomycin-induced SSc model mice. (C) Skin thickness from Control, PBS, hydrogel, MSCs, and Hy-MSCs groups mice after MSCs treatment. (D) Representative images of histopathological changes of Control, PBS, hydrogel, MSCs, and Hy-MSCs groups. The scale bar is 200 μm *P < 0.05, **P < 0.01.

The ability of MSCs-laden hydrogel to promote cell proliferation, retention time *in vivo*, cell migration, and vascularization *in vitro* indicates an improving clinical function. A murine skin fibrosis model by daily bleomycin injections were used in this study and were treated with PBS, hydrogel, MSCs, and MSCs-laden hydrogel (Hy-MSCs), respectively, as shown with the diagram in Fig. 6A. Body weight is important to evaluate the therapeutic efficacy. Compared to the control, bleomycin-induced SSc mice exhibited a weight loss at day 28, the body weight in Hy-MSCs and Hy-MSCs treated groups were significantly increased at day 40 (Fig. 6B). In addition, skin thickness was evaluated, which could reliably predict fibrotic changes in skin. It was found that significantly reduced skin thickness could be observed in MSCs and Hy-MSCs treated groups (Fig. 6C), indicating that the bleomycin‑induced SSc mice model was successfully established and MSCs and Hy-MSCs treatment could reduce the skin thickness.Furthermore, histological sections were examined. Compared with the control group, bleomycin administration led to remarkable collagen deposition as shown by Masson trichrome staining. A significant improvement in skin lesions was observed after injection of MSCs and Hy-MSCs, which was related to the marked decrease in collagen deposition, as showed in Fig. 6D.

**Fig. 7:**
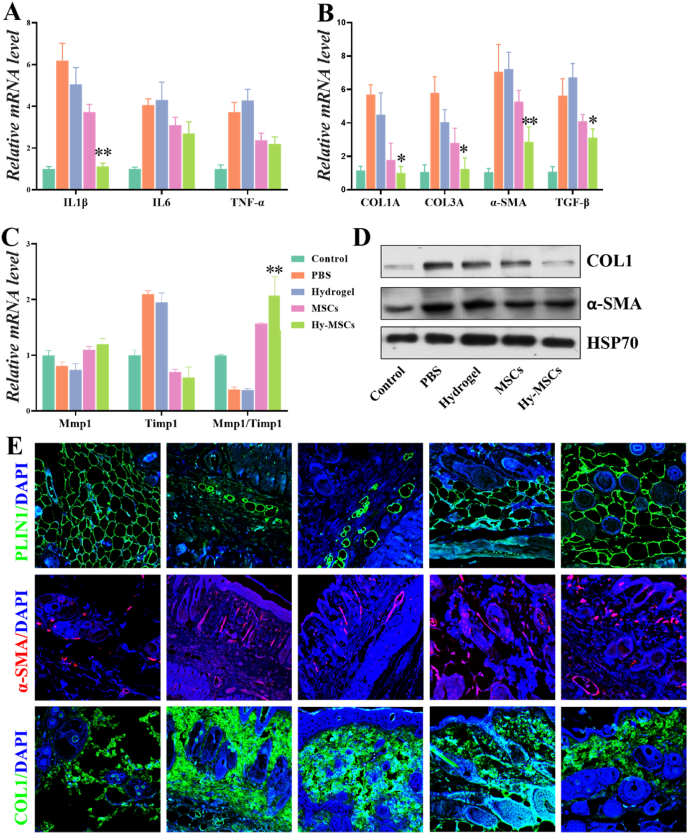
Comparative studies of anti-fibrotic therapeutic efficacy in bleomycin-induced SSc model mice. mRNA expression of cytokines including IL1β, IL6 and TNF-a (A), fibrotic markers including Col1A, Col3A, a-SMA and TGF-β (B), and remodeling parameters including Mmp1, Timp1 and Mmp/Timp1 (C) in skin sections from Control, PBS, hydrogel, MSCs, and Hy-MSCs groups of bleomycin-induced SSc model mice. (D) Western blot analysis of COL1 and a-SMA protein levels in skin tissues of Control, PBS, hydrogel, MSCs, and Hy-MSCs groups. HSP70 served as loading control. (E) Representative images of Immunofluorescence staining with PLIN, α-SMA and COL1 in skin sections from Control, PBS, hydrogel, MSCs, and Hy-MSCs groups of bleomycin-induced SSc model mice. The scale bar is 200 μm *P < 0.05, **P < 0.01.

To further investigate the anti-fibrosis effects of MSCs treatment, we evaluated the levels of inflammatory cytokines in impaired skin. Compared with control group, the levels of IL1β, IL6 and TNF-α in the skin were significantly increased in PBS and hydrogel group (Fig. 7A). MSCs-treatment significantly reduced the levels of IL1β, IL6 and TNF-α. The level of IL1β was reduced significantly in the Hy-MSCs treated group compared with that of MSCs group, while a slight decrease in the level of IL6 and TNF-α was noticed in Hy-MSCs group. These data indicated that the levels of inflammatory cytokines only partially contributed to the efficacy of MSCs treatment. Furthermore, the role of MSCs injection in collagen synthesis was then evaluated. As Col1A, Col3A, α-SMA and TGF-β are important genes associated with collagen synthesis, we analyzed the expressions of Col1A, Col3A, α-SMA and TGF-β in skin by qRT-PCR. As Fig. 7B showed, in comparison with the control group, bleomycin resulted in a significantly higher transcriptional expression of Col1A, Col3A, α-SMA and TGF-β in the skin. Notably, MSCs injection inhibited the gene expression levels of Col1A, Col3A, α-SMA and TGF-β compared with the PBS group. Tissue remodeling parameters were also affected by treatment, as illustrated by significant up-regulation of the expression of Mmp1/Timp1 ratio in MSCs treated groups (Fig. 7C). As expected, MSCs injection could significantly improve fibrosis by reducing collagen synthesis not only at mRNA levels but also at protein levels which were detected by Western blot and immunofluorescence technique (Fig. 7D and E). We found that there was a difference in collagen synthesis between MSCs and Hy-MSCs group. MSCs-loaded hydrogel infusion offered an approach to overcome the short persistence time of the stem cells, and control cell state and function. Therefore, Hy-MSCs injection led to a significant decrease in collagen synthesis related factors at mRNA and protein levels compared to MSCs group. As SSc also affected underlying fat tissue, Perilipin 1 (PLIN1), a crucial lipid droplet protein which can be detected in adipose tissue of the five groups were observed. We showed that MSCs treatment resumed PLIN1 expression level to the that of the control group (Fig. 7E). Therefore, these results showed that Hy-MSCs infusion reduced a fibrotic effect on a bleomycin-induced SSc. Taken together, these experimental results demonstrated that MSCs loaded hydrogel is an ideal strategy for treating SSc and a variety of clinical diseases.

## 4 Conclusion

In summary, we developed a new injectable MSCs-laden hydrogel for SSc treatment. This hydrogel system was engineered via the reversible Schiff base between the benzaldehyde at PEG-BA and amino groups on CS. Based on the dynamic chemistry of acylhydrazone bonds, the hydrogel showed fascinating injectable and self-healing capacities as well as attractive biocompatibility, degradation and non-toxicity property, making it a promising delivery of stem cell. MSCs in hydrogel could not only significantly promote cell proliferation, retention time *in vivo* but also improve their immune modulating functions, cell migration and vascularization *in vitro*. Notably, MSCs-laden hydrogel inhibited fibrosis by modulating the synthesis of collagen, anti-inflammatory and tissue remodeling and ameliorated disease progression in bleomycin induced SSc disease model mice after subcutaneous injection. These features indicate that MSCs-laden hydrogel is efficient and versatile for SSc, and could be used widely in the clinic.

## Declaration of competing interest

The authors declared that they have no conflicts of interest to this work.

The authors declared that they have no conflicts of interest to this work.

We declare that we do not have any commercial or associative interest that represents a conflict of interest in connection with the work submitted.

## CRediT authorship contribution statement

**Min Nie:** Formal analysis, Writing – original draft, carried out the experiments, analyzed data and wrote the paper. **Bin Kong:** Formal analysis, Writing – original draft, analyzed data and wrote the paper. **Guopu Chen:** Formal analysis, Writing – original draft, analyzed data and wrote the paper. **Ying Xie:** Writing – original draft, revised the manuscript and contributed to scientific discussion of the article. **Yuanjin Zhao:** Formal analysis, Writing – original draft, conceived the idea and designed the experiment, analyzed data and wrote the paper. **Lingyun Sun:** conceived the idea and designed the experiment.

## References

[1] Denton C.P., Khanna D. (2017). Systemic sclerosis. Lancet.

[2] Plikus M.V., Wang X., Sinha S., Forte E., Thompson S.M., Herzog E.L., Driskell R.R., Rosenthal N., Biernaskie J., Horsley V. (2021). Fibroblasts: origins, definitions, and functions in health and disease. Cell.

[3] Fugger L., Jensen L.T., Rossjohn J. (2020). Challenges, progress, and prospects of developing therapies to treat autoimmune diseases. Cell.

[4] Volkmann E.R., Varga J. (2019). Emerging targets of disease-modifying therapy for systemic sclerosis. Nat. Rev. Rheumatol..

[5] Fernandez-Codina A., Walker K.M., Pope J.E. (2018). Treatment algorithms for systemic sclerosis according to experts. Arthritis Rheumatol..

[6] Volkmann E.R., Tashkin D.P., Sim M., Li N., Goldmuntz E., Keyes-Elstein L., Pinckney A., Furst D.E., Clements P.J., Khanna D., Steen V., Schraufnagel D.E., Arami S., Hsu V., Roth M.D., Elashoff R.M., Sullivan K.M. (2019). Short-term progression of interstitial lung disease in systemic sclerosis predicts long-term survival in two independent clinical trial cohorts. Ann. Rheum. Dis..

[7] Zhang H., Liang J., Tang X., Wang D., Feng X., Wang F., Hua B., Wang H., Sun L. (2017). Sustained benefit from combined plasmapheresis and allogeneic mesenchymal stem cells transplantation therapy in systemic sclerosis. Arthritis Res. Ther..

[8] Kowal-Bielecka O., Fransen J., Avouac J., Becker M., Kulak A., Allanore Y., Distler O., Clements P., Cutolo M., Czirjak L., Damjanov N., Del G.F., Denton C.P., Distler J., Foeldvari I., Figelstone K., Frerix M., Furst D.E., Guiducci S., Hunzelmann N., Khanna D., Matucci-Cerinic M., Herrick A.L., van den Hoogen F., van Laar J.M., Riemekasten G., Silver R., Smith V., Sulli A., Tarner I., Tyndall A., Welling J., Wigley F., Valentini G., Walker U.A., Zulian F., Muller-Ladner U. (2017). Update of EULAR recommendations for the treatment of systemic sclerosis. Ann. Rheum. Dis..

[9] Henderson J., Distler J., O'Reilly S. (2019). The role of epigenetic modifications in systemic sclerosis: a druggable target. Trends Mol. Med..

[10] van Bijnen S., de Vries-Bouwstra J., van den Ende C.H., Boonstra M., Kroft L., Geurts B., Snoeren M., Schouffoer A., Spierings J., van Laar J.M., Huizinga T.W., Voskuyl A., Marijt E., van der Velden W., van den Hoogen F.H., Vonk M.C. (2020). Predictive factors for treatment-related mortality and major adverse events after autologous haematopoietic stem cell transplantation for systemic sclerosis: results of a long-term follow-up multicentre study. Ann. Rheum. Dis..

[11] Pittenger M.F., Discher D.E., Peault B.M., Phinney D.G., Hare J.M., Caplan A.I. (2019). Mesenchymal stem cell perspective: cell biology to clinical progress. NPJ Regen. Med..

[12] Galipeau J., Sensebe L. (2018). Mesenchymal stromal cells: clinical challenges and therapeutic opportunities. Cell Stem Cell.

[13] Song N., Scholtemeijer M., Shah K. (2020). Mesenchymal stem cell immunomodulation: mechanisms and therapeutic potential. Trends Pharmacol. Sci..

[14] Shi Y., Du L., Lin L., Wang Y. (2017). Tumour-associated mesenchymal stem/stromal cells: emerging therapeutic targets. Nat. Rev. Drug Discov..

[15] Andrzejewska A., Lukomska B., Janowski M. (2019). Concise review: mesenchymal stem cells: from roots to boost. Stem Cell..

[16] El A.E., Kramann R., Schneider R.K., Li X., Seeger W., Humphreys B.D., Bellusci S. (2017). Mesenchymal stem cells in fibrotic disease. Cell Stem Cell.

[17] Shi Y., Wang Y., Li Q., Liu K., Hou J., Shao C., Wang Y. (2018). Immunoregulatory mechanisms of mesenchymal stem and stromal cells in inflammatory diseases. Nat. Rev. Nephrol..

[18] Koliaraki V., Prados A., Armaka M., Kollias G. (2020). The mesenchymal context in inflammation, immunity and cancer. Nat. Immunol..

[19] Naik S., Larsen S.B., Cowley C.J., Fuchs E. (2018). Two to Tango: dialog between immunity and stem cells in health and disease. Cell.

[20] Ridge S.M., Sullivan F.J., Glynn S.A. (2017). Mesenchymal stem cells: key players in cancer progression. Mol. Cancer.

[21] Rozier P., Maumus M., Maria A., Toupet K., Lai-Kee-Him J., Jorgensen C., Guilpain P., Noel D. (2021). Mesenchymal stromal cells-derived extracellular vesicles alleviate systemic sclerosis via miR-29a-3p. J. Autoimmun..

[22] Linthout S.V., Spillmann F., Schultheiss H.P., Tschope C. (2011). Effects of mesenchymal stromal cells on diabetic cardiomyopathy. Curr. Pharmaceut. Des..

[23] Maria A.T., Toupet K., Bony C., Pirot N., Vozenin M.C., Petit B., Roger P., Batteux F., Le Quellec A., Jorgensen C., Noel D., Guilpain P. (2016). Antifibrotic, antioxidant, and immunomodulatory effects of mesenchymal stem cells in HOCl-induced systemic sclerosis. Arthritis Rheumatol..

[24] Mao A.S., Ozkale B., Shah N.J., Vining K.H., Descombes T., Zhang L., Tringides C.M., Wong S.W., Shin J.W., Scadden D.T., Weitz D.A., Mooney D.J. (2019). Programmable microencapsulation for enhanced mesenchymal stem cell persistence and immunomodulation. Proc. Natl. Acad. Sci. U. S. A..

[25] Ankrum J.A., Ong J.F., Karp J.M. (2014). Mesenchymal stem cells: immune evasive, not immune privileged. Nat. Biotechnol..

[26] Zhang Y.S., Khademhosseini A. (2017). Advances in engineering hydrogels. Science.

[27] Khodambashi R., Alsaid Y., Rico R., Marvi H., Peet M.M., Fisher R.E., Berman S., He X., Aukes D.M. (2021). Heterogeneous hydrogel structures with spatiotemporal reconfigurability using addressable and tunable voxels. Adv. Mater..

[28] Fu F., Shang L., Chen Z., Yu Y., Zhao Y. (2018). Bioinspired living structural color hydrogels. Sci Robot.

[29] Sun L., Yu Y., Chen Z., Bian F., Ye F., Sun L., Zhao Y. (2020). Biohybrid robotics with living cell actuation. Chem. Soc. Rev..

[30] Wang Y., Shang L., Chen G., Sun L., Zhang X., Zhao Y. (2020). Bioinspired structural color patch with anisotropic surface adhesion. Sci. Adv..

[31] Yan B., Huang J., Han L., Gong L., Li L., Israelachvili J.N., Zeng H. (2017). Duplicating dynamic strain-stiffening behavior and nanomechanics of biological tissues in a synthetic self-healing flexible network hydrogel. ACS Nano.

[32] Miri A.K., Nieto D., Iglesias L., Goodarzi H.H., Maharjan S., Ruiz-Esparza G.U., Khoshakhlagh P., Manbachi A., Dokmeci M.R., Chen S., Shin S.R., Zhang Y.S., Khademhosseini A. (2018). Microfluidics-enabled multimaterial maskless stereolithographic bioprinting. Adv. Mater..

[33] Liu Y., Du J., Peng P., Cheng R., Lin J., Xu C., Yang H., Cui W., Mao H., Li Y., Geng D. (2021). Regulation of the inflammatory cycle by a controllable release hydrogel for eliminating postoperative inflammation after discectomy. Bioact. Mater..

[34] Lee A.S., Inayathullah M., Lijkwan M.A., Zhao X., Sun W., Park S., Hong W.X., Parekh M.B., Malkovskiy A.V., Lau E., Qin X., Pothineni V.R., Sanchez-Freire V., Zhang W.Y., Kooreman N.G., Ebert A.D., Chan C., Nguyen P.K., Rajadas J., Wu J.C. (2018). Prolonged survival of transplanted stem cells after ischaemic injury via the slow release of pro-survival peptides from a collagen matrix. Nat. Biomed. Eng..

[35] Mao A.S., Shin J.W., Utech S., Wang H., Uzun O., Li W., Cooper M., Hu Y., Zhang L., Weitz D.A., Mooney D.J. (2017). Deterministic encapsulation of single cells in thin tunable microgels for niche modelling and therapeutic delivery. Nat. Mater..

[36] Huang Q., Zou Y., Arno M.C., Chen S., Wang T., Gao J., Dove A.P., Du J. (2017). Hydrogel scaffolds for differentiation of adipose-derived stem cells. Chem. Soc. Rev..

[37] A S., Lyu J., Johnson M., Creagh-Flynn J., Zhou D., Lara-Saez I., Xu Q., Tai H., Wang W. (2020). Instant gelation system as self-healable and printable 3D cell culture bioink based on dynamic covalent chemistry. ACS Appl. Mater. Interfaces.

[38] Yan B., Han L., Xiao H., Zhang J., Huang J., Hu W., Gu Y., Liu Q., Zeng H. (2019). Rapid dewatering and consolidation of concentrated colloidal suspensions: mature fine tailings via self-healing composite hydrogel. ACS Appl. Mater. Interfaces.

[39] Chen H., Jia P., Kang H., Zhang H., Liu Y., Yang P., Yan Y., Zuo G., Guo L., Jiang M., Qi J., Liu Y., Cui W., Santos H.A., Deng L. (2016). Upregulating hif-1alpha by hydrogel nanofibrous scaffolds for rapidly recruiting angiogenesis relative cells in diabetic wound. Adv. Health Mater..

[40] Mao X., Cheng R., Zhang H., Bae J., Cheng L., Zhang L., Deng L., Cui W., Zhang Y., Santos H.A., Sun X. (2019). Self-healing and injectable hydrogel for matching skin flap regeneration. Adv. Sci..

[41] Yang X., Yang H., Jiang X., Yang B., Zhu K., Lai N.C., Huang C., Chang C., Bian L., Zhang L. (2021). Injectable chitin hydrogels with self-healing property and biodegradability as stem cell carriers. Carbohydr. Polym..

[42] Zou W., Chen Y., Zhang X., Li J., Sun L., Gui Z., Du B., Chen S. (2018). Cytocompatible chitosan based multi-network hydrogels with antimicrobial, cell anti-adhesive and mechanical properties. Carbohydr. Polym..

[43] Chi J., Zhang X., Chen C., Shao C., Zhao Y., Wang Y. (2020). Antibacterial and angiogenic chitosan microneedle array patch for promoting wound healing. Bioact. Mater..

[44] Xin Y., Yuan J. (2012). Schiff's base as a stimuli-responsive linker in polymer chemistry. Polym. Chem..

[45] Madl C.M., Heilshorn S.C., Blau H.M. (2018). Bioengineering strategies to accelerate stem cell therapeutics. Nature.

[46] Zhu Z., Guo S.Z., Hirdler T., Eide C., Fan X., Tolar J., McAlpine M.C. (2018). 3D printed functional and biological materials on moving freeform surfaces. Adv. Mater..

[47] Zhang K., Jia Z., Yang B., Feng Q., Xu X., Yuan W., Li X., Chen X., Duan L., Wang D., Bian L. (2018). Adaptable hydrogels mediate cofactor-assisted activation of biomarker-responsive drug delivery via positive feedback for enhanced tissue regeneration. Adv. Sci..

[48] Qin M., Sun M., Bai R., Mao Y., Qian X., Sikka D., Zhao Y., Qi H.J., Suo Z., He X. (2018). Bioinspired hydrogel interferometer for adaptive coloration and chemical sensing. Adv. Mater..

[49] Zou W., Chen Y., Zhang X., Li J., Sun L., Gui Z., Du B., Chen S. (2018). Cytocompatible chitosan based multi-network hydrogels with antimicrobial, cell anti-adhesive and mechanical properties. Carbohydr. Polym..

[50] Huang W., Wang Y., Chen Y., Zhao Y., Zhang Q., Zheng X., Chen L., Zhang L. (2016). Strong and rapidly self-healing hydrogels: potential hemostatic materials. Adv. Health Mater..

[51] Koh J., Griffin D.R., Archang M.M., Feng A.C., Horn T., Margolis M., Zalazar D., Segura T., Scumpia P.O., Di Carlo D. (2019). Enhanced in vivo delivery of stem cells using microporous annealed particle scaffolds. Small.

[52] Zhang Y.S., Khademhosseini A. (2017). Advances in engineering hydrogels. Science.

[53] Galipeau J., Sensebe L. (2018). Mesenchymal stromal cells: clinical challenges and therapeutic opportunities. Cell Stem Cell.

