# Supplementary material for: MSCs-laden injectable self-healing hydrogel for systemic sclerosis treatment
Source: Bioact Mater. 2022 Jan 19;17:369–78. doi: 10.1016/j.bioactmat.2022.01.006 (PMC8964965; doi:10.1016/j.bioactmat.2022.01.006)
Supplement: Multimedia Component 1 [file mmc1.docx]

**Supporting information**

**MSCs-laden injectable self-healing hydrogel for systemic sclerosis treatment**

**Min Nie^1^, Bin Kong^2^, Guopu Chen^1^, Ying Xie^3^, Yuanjin Zhao^1,4^, Lingyun Sun^1,^ ***

^1^ Department of Rheumatology and Immunology, Institute of Translational Medicine, The Affiliated Drum Tower Hospital of Nanjing University Medical School, Nanjing, 210002, China

^2^ Department of Neurosurgery, Health Science Center, The First Affiliated Hospital of Shenzhen University, Shenzhen Second People's Hospital, Shenzhen, 518035, China

^3^ State Key Laboratory of Quality Research in Chinese Medicines, Macau University of Science and Technology, Taipa, Macau (SAR)

^4^ State Key Laboratory of Bioelectronics, School of Biological Science and Medical Engineering, Southeast University, Nanjing 210096, China

**^*^** Corresponding authors:

Email: [lingyunsun@nju.edu.cn](mailto:lingyunsun@nju.edu.cn) (Lingyun Sun)

**Table S1.** List of individual gene primers.

| Gene Forward Reverse |
| --- |
| Human-CCL2 AGGTGACTGGGGCATTGAT GCCTCCAGCATGAAAGTCTC  Human-COX-2 TGACCAGAGCAGGCAGATGAA CCACAGCATCGATGTCACCATAG  Human-TGF-β AGCGACTCGCCAGAGTGGTTA GCAGTGTGTTATCCCTGCTGTCA  Human-TSG-6 TCTGTGCTGCTGGATGGATG TCCTTTGCGTGTGGGTTGTA  Human-PGE-2 GTGACCGAGTTCGGCAATAAG CGGACAATGTAGTCAAAGGACG  Human-HMOX CTTCTTCACCTTCCCCAACA AGCTCCTGCAACTCCTCAAA  Human-HGF GCTATCGGGGTAAAGACCTACA CGTAGCGTACCTCTGGATTGC  Human-FGF-2 TCCACCTATAATTGGTCAAAGTGGT CATCAGTTACCAGCTCCCCC  Human-GAPDH CGAGCCACATCGCTCAGACA GTGGTGAAGACGCCAGTGGA  Mouse-TGF-β AGGGCTACCATGCCAACTTC CCACGTAGTAGACGATGGGC  Mouse-IL1β TGTGAAATGCCACCTTTTGA GGTCAAAGGTTTGGAAGCAG  Mouse-IL6 GATGGATGCTACCAAACTGGA TCTGAAGGACTCTGGCTTTG  Mouse-TNFα CAGGCGGTGCCTATGTCTC CGATCACCCCGAAGTTCAGTAG  Mouse-Col1A TAAAGGGTCATCGTGGCTTC GACGGCTGAGTAGGGAACAC  Mouse-Col3A CTGTAACATGAAACTGAACTGAAA CCATAGCTGAACTGAAAACCACC  Mouse-α-SMA CTGTCCCTCTATGCCTCTGG AGGGCTGTGATCTCCTTCTG  Mouse-Mmp1 CCTTGATGAGACGTGGACCAA ATGTGGTGTTGTTGCACCTGT  Mouse-Timp1 CTCCGCCCTTCGCATGGACATT GGGGGCCATCATGGTATCTGCTCT  Mouse-GAPDH AGGTCGGTGTGAACGGATTTG GGGGTCGTTGATGGCAACA |


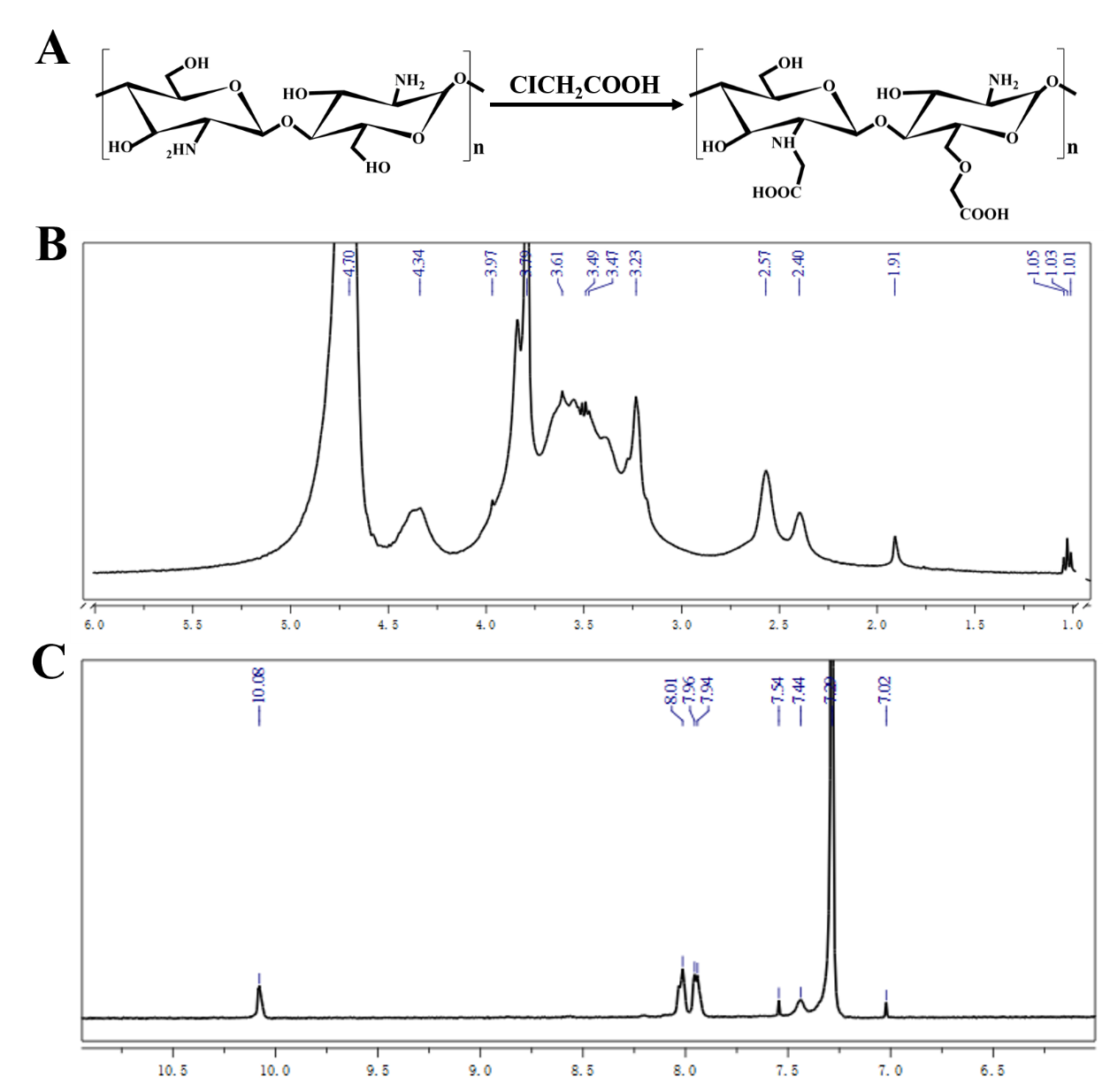


**Figure S1**. (A) Chemical synthesizing process of the PEGBA. (B) ^1^H NMR spectra of the CM-CS. The peaks of 3.2–3.5 ppm indicated the H-2 proton of carboxymethyl. (C) ^1^H NMR spectra of the PEG-BA. Aldehyde (10.08 ppm) and benzene ring (8.01 and 7.96 ppm) were observed.


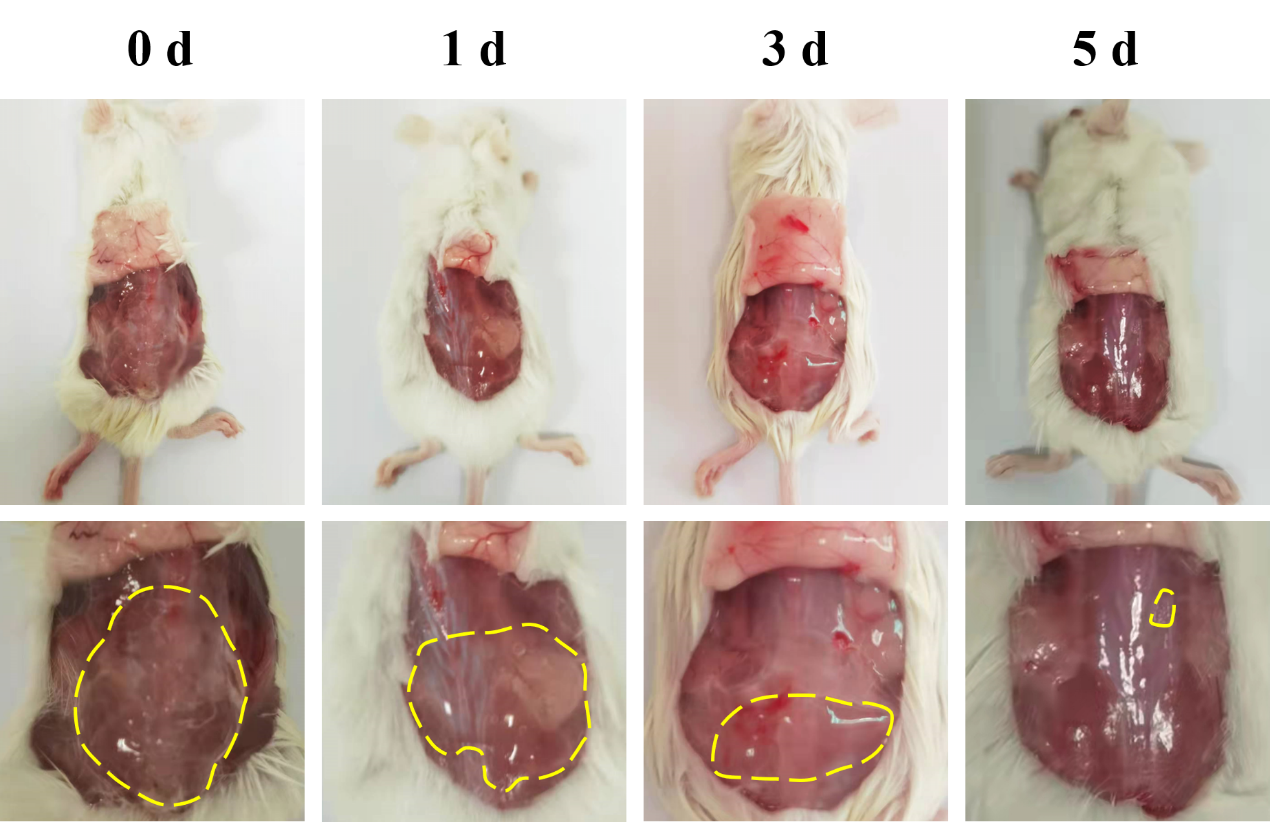


**Figure S2.** Observation of the degradation assay *in vivo* during different periods. The amount of hydrogel decreased gradually in the mouse body (indicated with yellow frames).


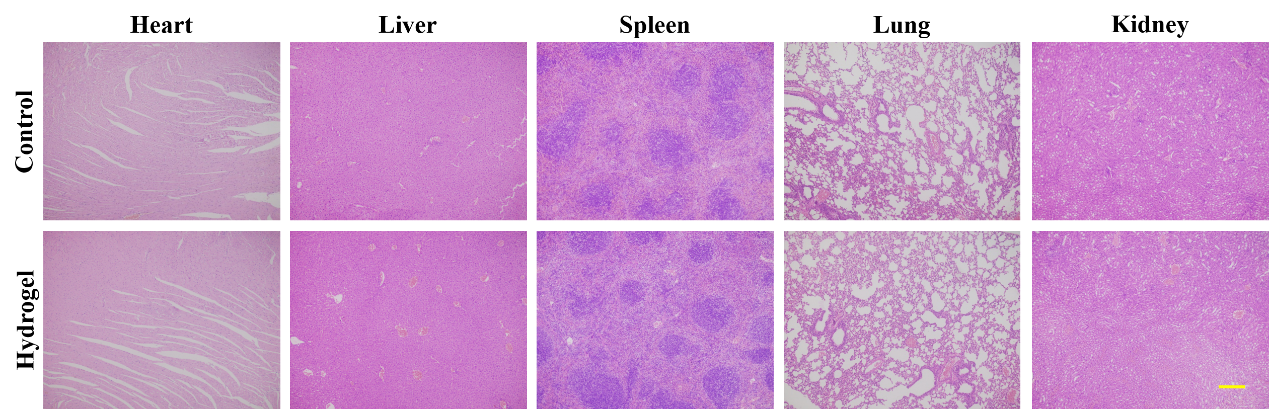


**Figure S3.** H&E stained organs revealed no significant signs of toxicity. The scale bar is 200 μm.


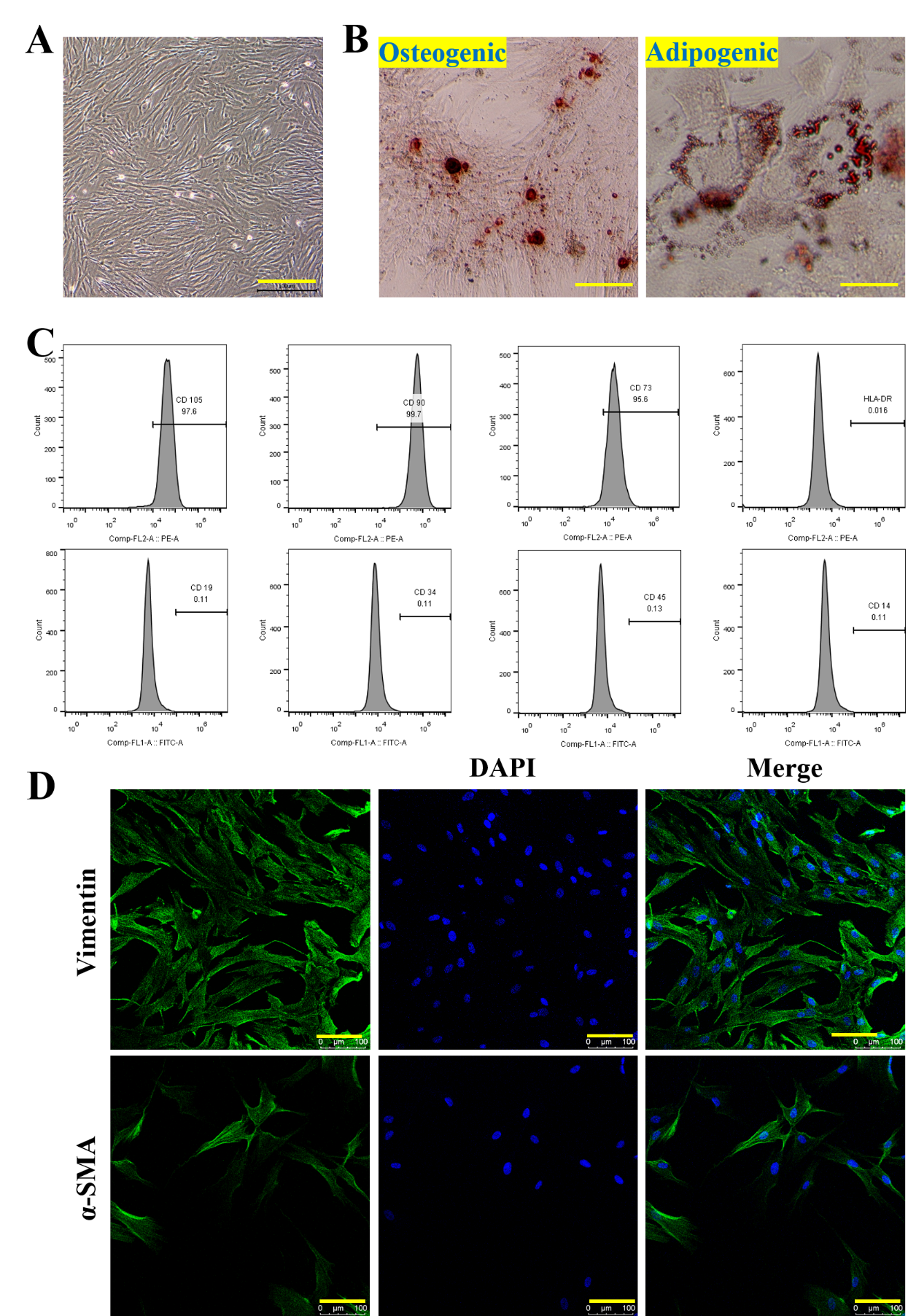


**Figure S4. Characteristics and differentiation potential of clinical-grade MSCs derived from primary human umbilical cord (**hUC-MSCs**). (A)** Representative optical morphological images of primary clinical-grade hUC-MSCs of P 3 derived from optimized tissue blocks. **(B)** Differentiation potential of hUC-MSCs into mesodermal lineages. Representative images of hUC-MSCs differentiated into osteocytes and adipocytes are shown as indicated. Calcium phosphate deposits were stained with Alizarin Red. Fat droplets were stained with Oil red O. **(C)** Flow cytometric analysis showed hUC-MSCs were positive for mesenchymal lineage markers (CD105, CD90 and CD73), negative for (HLA-DR, CD19, CD34, CD45 and CD14). **(D)** Immunofluorescence staining of hUC-MSCs showed they were positive for mesenchymal markers of Vimentin and α-SMA. The scale bar is 100 μm.


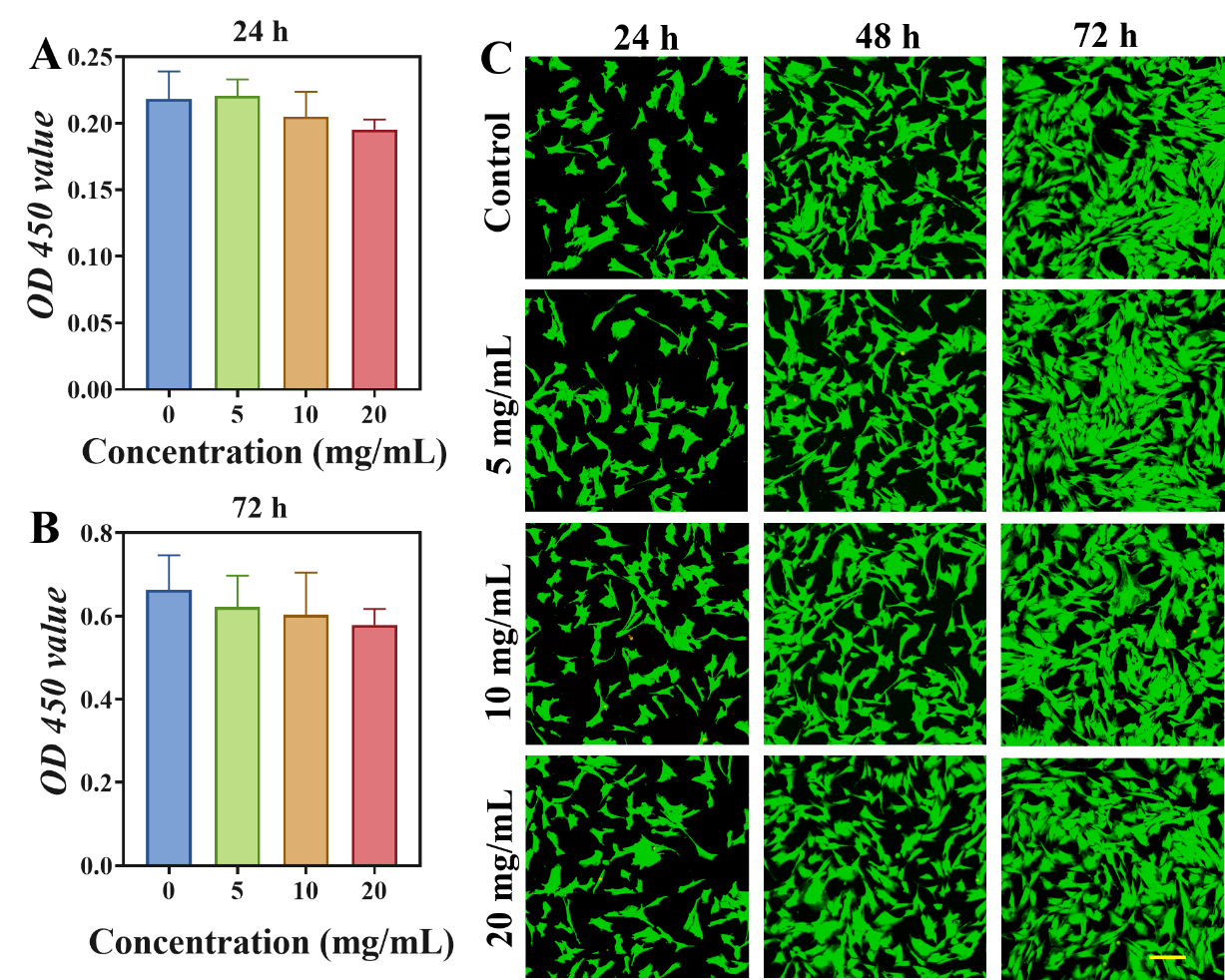


**Figure S5.** Cytocompatibility evolution of the hydrogels extracts against MSCs with different concentrations. The viability of MSCs exposed to hydrogel extracts with different concentrations for 24 h (A) and 72 h (B) was evaluated using CCK-8 assay. (C) Live/dead staining of MSCs after being incubated in hydrogel extracts with different concentrations for 24, 48, and 72 h. The scale bar is 200 μm.


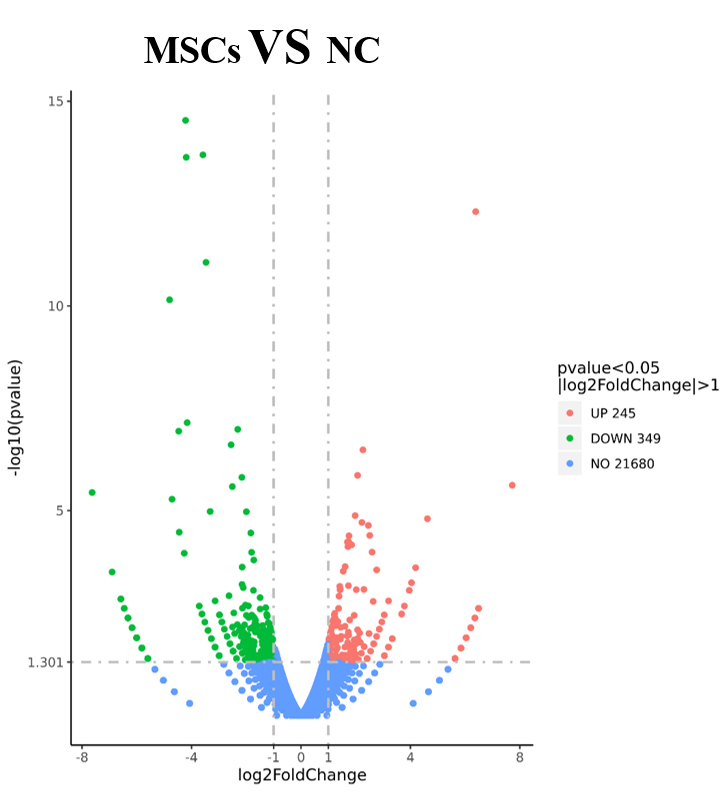


**Figure S6.** Differential gene statistics. The abscissa was Log2 Fold Change value, the ordinate was -Log10 (*p* value), and the dashed blue line represented the threshold line of the differential gene screening criteria.


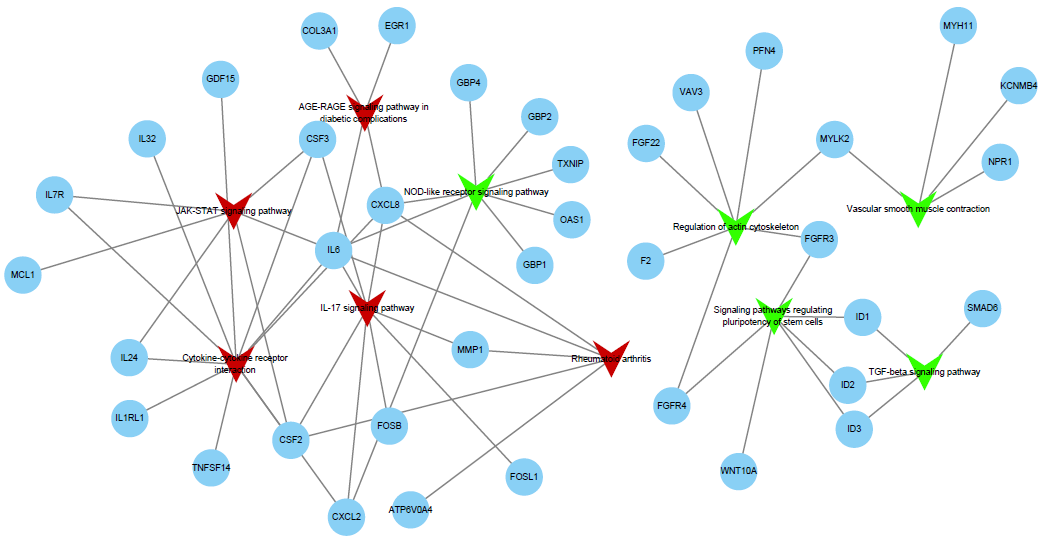


**Figure S7.** The network of KEGG pathways illustrating the interactions between the groups of genes
